# Perchlorate-Coupled Carbon Monoxide (CO) Oxidation: Evidence for a Plausible Microbe-Mediated Reaction in Martian Brines

**DOI:** 10.3389/fmicb.2017.02571

**Published:** 2017-12-22

**Authors:** Marisa R. Myers, Gary M. King

**Affiliations:** Department of Biological Sciences, Louisiana State University, Baton Rouge, LA, United States

**Keywords:** carbon monoxide, extreme halophile, perchlorate reduction, Mars, chlorate reduction

## Abstract

The presence of hydrated salts on Mars indicates that some regions of its surface might be habitable if suitable metabolizable substrates are available. However, several lines of evidence have shown that Mars’ regolith contains only trace levels of the organic matter needed to support heterotrophic microbes. Due to the scarcity of organic carbon, carbon monoxide (CO) at a concentration of about 700 parts per million (about 0.4 Pa) might be the single most abundant readily available substrate that could support near-surface bacterial activity. Although a variety of electron acceptors can be coupled to CO oxidation, perchlorate is likely the most abundant potential oxidant in Mars’ brines. Whether perchlorate, a potent chaotrope, can support microbial CO oxidation has not been previously documented. We report here the first evidence for perchlorate-coupled CO oxidation based on assays with two distinct euryarchaeal extreme halophiles. CO oxidation occurred readily in 3.8 M NaCl brines with perchlorate concentrations from 0.01 to 1 M. Both isolates were able to couple CO with perchlorate or chlorate under anaerobic conditions with or without nitrate as an inducer for nitrate reductase, which serves as a perchlorate reductase in extreme halophiles. In the presence of perchlorate, CO concentrations were reduced to levels well below those found in Mars’ atmosphere. This indicates that CO could contribute to the survival of microbial populations in hydrated salt formations or brines if water activities are suitably permissive.

## Introduction

The prospects for extraterrestrial life depend on two major factors: liquid water availability and the availability of reduced inorganic and organic substrates that can be used to sustain biochemical reactions (Brack et al., 2010). While discoveries of liquid water on Europa and Enceladus have addressed the former (Kargel et al., 2000; Zolotov et al., 2004; Zolotov, 2007; Waite et al., 2009; Ojha et al., 2014, 2015), much speculation remains about the latter. For Europa and Enceladus, simple organic and inorganic compounds have been identified as substrates for putative anaerobic processes (Chyba, 2000; Chyba and Phillips, 2002; McKay et al., 2008; Waite et al., 2017). Some of these substrates, especially hydrogen and formate, might be produced by serpentinization or other reactions beneath or associated with ice-capped oceans (Chyba, 2000). Evidence for liquid water has also been reported on Mars (Ojha et al., 2014, 2015), though Dundas et al. (2017) have indicated that volumes are likely low. Regardless, suites of organics at substrate level concentrations have not yet been identified in the regolith (Benner et al., 2000). Although hydrothermal activity and serpentinization might occur in the deep sub-surface of Mars, these processes are unlikely to contribute reactants to surface brines that might host microbes.

However, in contrast to Europa and Enceladus, Mars’ atmosphere serves as a reservoir for carbon monoxide (CO), a relatively abundant [about 700 parts per million (ppm), 0.4 Pa] photochemically produced reductant (Sindoni et al., 2010; Mahaffy et al., 2013) that can serve as a microbial metabolite under extreme conditions (King, 2015). Indeed, based on the total mass of Mars’ atmosphere, its average molar mass (43.34 g mol^-1^) and a concentration of 700 ppm, CO carbon occurs at 2.8 mol m^-2^ assuming a uniform distribution across the regolith surface. This is equivalent to the amount of carbon in 1 m^3^ of regolith with a density of 2 g cm^-3^ and a carbon concentration of 17 ppm. Since total organic carbon ranges from about 10 to 500 ppm for meteorites derived from Mars (Steele et al., 2012) and Mars mudstones (Ming et al., 2014; Freissinet et al., 2015), CO might be the single most abundant readily available and renewable resource to drive metabolic activity.

Although diverse lineages of terrestrial bacteria can couple CO to multiple electron acceptors [e.g., molecular oxygen, nitrate, and sulfate (Ragsdale, 2004; King, 2006; King and Weber, 2007)], hydrated salts and brines on Mars are likely dominated by perchlorate and chlorate (Hecht et al., 2009; Kounaves et al., 2014; Clark and Kounaves, 2016), which have not been shown to support CO oxidation. However, both oxyanions support metabolism of various organic substrates by bacteria and archaea (Coates and Achenbach, 2004; Liebensteiner et al., 2013; Oren et al., 2014; Martinez-Espinosa et al., 2015; Mehta-Kolte et al., 2017) that reduce perchlorate via a dissimilatory perchlorate reductase (only bacteria to date) or a dissimilatory nitrate reductase (bacteria and archaea). To date, no CO-oxidizing dissimilatory perchlorate-reducing bacteria have been isolated, but numerous denitrifying and nitrate-respiring CO oxidizers have been reported (King, 2006, 2015; Weber and King, 2017). Results presented here provide the first evidence that some denitrifying and nitrate-respiring euryarchaeal extreme halophiles can couple CO oxidation in brines to perchlorate at concentrations up to 1 M.

## Materials and Methods

### Isolates

Briefly, several denitrifying or nitrate-respiring CO-oxidizing extremely halophilic euryarchaeotes were obtained from salt crusts or soils in or near the Bonneville Salt Flats (BSF; Utah, United States). One of the denitrifiers, *Haloarcula* sp. PCN7, was obtained from enrichments initiated with BSF salt crusts (40° 45′ 26.4″, -113° 53′ 11.1″) while the nitrate-respiring *Halobaculum* sp. WSA2 was obtained from enrichments of a saline soil collected south of the BSF (40° 25′ 43.0″, -114° 00′ 55.4″). Enrichments were conducted in 160-ml serum bottles under anoxic (nitrogen headspace with nitrate as an electron acceptor for *Haloarcula* sp. PCN7) or oxic conditions (for *Halobaculum* sp. WSA2) in medium CM1 (McDuff et al., 2016) with 25 mM pyruvate. After addition of approximately 100 ppm CO (final concentration), serum bottles were incubated at 40°C with shaking. Headspace CO was monitored by gas chromatographic analysis (McDuff et al., 2016). Enrichments positive for CO oxidation were used to inoculate a series of bottles with fresh CM1 media, which were ultimately used to prepare dilutions that were spread onto CM1-pyruvate agar plates. Distinct colonies were selected and purified through repeated sub-culturing as necessary. Isolates were identified to genus by PCR amplification and sequencing of 16S rRNA genes obtained from genomic extracts using a MoBio Microbial DNA Extraction Kit (Folsom, CA, United States). PCR used an archaeal forward primer, Arch21F [5′-TTCCGGTTGATCCYGCCGGA-3′ (Delong, 1992)], and the universal reverse primer, 1492R [5′-CGGTTACCTTGTTACGACTT-3′ (Lane, 1991)]. Purified amplicons (MoBio Ultraclean PCR Clean-Up Kit) were sequenced with an ABI 3130XL Genetic Analyzer at Louisiana State University. Sequences have been deposited in Genbank as accessions MF767880 and MF767881 for *Haloarcula* sp. PCN7 and *Halobaculum* sp. WSA2, respectively.

Genomic DNA extracts were also used to amplify the alpha sub-unit (*narG*) of nitrate reductase (Martinez-Espinosa et al., 2007, 2015) and the large sub-unit (*coxL*) of the form I CO dehydrogenase (King, 2003). Primers for *narG* were designed using nitrate reductase sequences found in the genomes of *Haloarcula marismortui* 43049^T^, *Haloferax mediterranei* 33500^T^, and other haloarchaea represented in the Integrated Microbial Genomes resource^1^. Primers pNar1F (5′-ACGAYTGGTAYCACAACGAC-3′) and pNar1R (5′-AGTTCSAGRWACCAGTCGTG-3′) yield products approximately 990 bp in size. Details of *coxL* PCR have been published previously (King, 2003); PCR primers used were archcoxF (5′-GGYGGSTTYGGSAASAAGGT-3′) and PSr (5′-YTCGAYGATCATCGGRTTGA-3′). Amplicons obtained using these primers were sequenced bi-directionally as above. Phylogenetic analyses of inferred amino acid sequences for *coxL* and *narG* were conducted using MEGA7 (Kumar et al., 2015) using a neighbor-joining algorithm and a Poisson correction; all gapped positions were deleted. *CoxL* sequences have been deposited in Genbank as accessions MF773971 and MF773972 for *Haloarcula* sp. PCN7 and *Halobaculum* sp. WSA2, respectively, and as MF773973 and MF773974 for the respective *narG* genes.

Assays for nitrate respiration and denitrification were conducted using API 20NE test strips (bioMérieux S.A., Marcy l’Etoile, France) according to the manufacturer’s instructions. Confirmation of the results for *Haloarcula* sp. PCN7 was obtained by incubating the isolate in sealed 10-cm^3^ syringes with CM1 medium containing pyruvate and nitrate. Under these conditions, copious production of gas bubbles presumed to be dinitrogen was interpreted as evidence for denitrification.

Although both *Haloarcula* sp. PCN7 and *Halobaculum* sp. WSA2 can utilize CO at concentrations in excess of 100 ppm, growth was not observed for either isolate. This is consistent with results obtained for other CO-oxidizing extreme halophiles (McDuff et al., 2016).

### Perchlorate-Coupled CO Oxidation

*Haloarcula* sp. PCN7 and *Halobaculum* sp. WSA2 were assessed for their ability to couple CO oxidation to perchlorate reduction using stationary phase cells first grown aerobically in 3.8 M CM1-pyruvate medium to provide a suitable level of cell biomass. Since CO uptake is typically induced by substrate limitation during stationary phase, it was not necessary to pre-incubate cells with CO. To initiate assays, cells were harvested by centrifugation, washed in 3.8 M CM1 without pyruvate, and resuspended into 3.8 M CM1 with or without 2.5 mM pyruvate to support basal metabolism; treatments included the following: aerobic incubation with no perchlorate; anaerobic incubation (nitrogen headspace) with 0.01 M perchlorate with or without 0.25 mM nitrate; anaerobic incubation with no electron acceptor and anaerobic incubation of autoclaved cells with 0.01 M perchlorate. Resuspended cells (10 ml, *A*_600_ ∼ 0.4) were incubated in triplicate 160-ml serum bottles sealed with neoprene rubber stoppers. CO was added to a final headspace (150 ml) concentration of ∼10 ppm (about 1 Pa). Headspace CO concentrations were assayed periodically by removing sub-samples for analysis by gas chromatography. Anoxic treatments contained resazurin to monitor oxygen contamination.

To assess tolerance of elevated perchlorate concentrations cells were grown to stationary phase, centrifuged, and washed as described above. Resuspended cells were aliquoted into 160-ml serum bottles to which either 0.01, 0.1, or 1 M perchlorate was added; each perchlorate treatment was incubated with aerobic (21% oxygen) or anoxic (flushed with nitrogen) headspaces. Experimental treatments without perchlorate included oxic, no electron acceptor, and an autoclaved kill control. All treatments were performed in triplicate.

Perchlorate concentrations were determined periodically during selected treatments using sub-samples obtained by needle and syringe. Perchlorate concentrations were measured with an ion-selective electrode (Thomas Scientific, Swedesboro, NJ, United States), standardized with perchlorate dissolved in growth medium. Total assay volume was 5 m1 including 500 μ1 of culture and 100 μl of 1 M sodium acetate as an ionic strength adjustment buffer. A 10-fold sample dilution was required due to the high NaCl concentrations in the media.

Chlorate concentrations were monitored via a colorimetric *O*-tolidine assay (Couture, 1998). *O*-Tolidine assays contained in a 1-ml final reaction mixture: 4 μl sample, 396 μl deionized water, 100 μl *O*-tolidine, and 500 μl concentrated (12 M) HCl. After 10 min of incubation, absorbance was read at 448 nm.

Water potentials of growth media were assessed using a WP4-T water potential meter [Decagon Devices, Pullman, WA, United States (Weber and King, 2009)]. Water potential (with units of pressure, e.g., MPa) is a measure of the chemical potential of water molecules in solutions, and varies with temperature and solute concentration and composition (King, 2017). Pure water has a potential of 0 while solutions have potentials <0 with decreasing values representing increasing physiological water stress. For reference, seawater containing 3.5% NaCl has a water potential of about -2.8 MPa and NaCl saturated brines have potentials about -40 MPa (King, 2015). The water potentials of CM1 media with varied perchlorate concentration were measured to account for variations in water stresses.

## Results and Discussion

### Perchlorate Linked CO Oxidation by a Denitrifying Extreme Halophile

To assess the feasibility of CO-coupled perchlorate reduction in brines, denitrifying and nitrate-respiring extremely halophilic enrichments and isolates were obtained from the BSF and nearby saline soils (UT, United States). Previous reports have shown that a dissimilatory periplasmic nitrate reductase catalyzes perchlorate, chlorate, and nitrate reduction in the denitrifying extreme halophile, *Hfx. mediterranei* 33500^T^, with chlorate reduction rates exceeding those for nitrate (Martinez-Espinosa et al., 2015). However, there has been no evidence to date for dissimilatory perchlorate-reducing extreme halophiles.

*Haloarcula* sp. PCN7 was selected as a model denitrifier for assays with perchlorate. It oxidizes CO in media with up to 5.2 M NaCl (halite saturation) using molecular oxygen as an electron acceptor; it also oxidizes CO using nitrate during denitrification. It possesses a canonical molybdenum-dependent form I CO dehydrogenase (Supplementary Figure 1), and it contains a nitrate reductase gene as established by PCR amplification of the *narGH* structural gene and genomic analysis. Sequence and phylogenetic analyses have revealed characteristic motifs for dissimilatory nitrate reductases (Martinez-Espinosa et al., 2007) and a nucleotide identity of 80.3% with the *Hfx. mediterranei* 33500^T^ *narGH* gene (Supplementary Figure 2).

Since CO uptake was typically assayed with initial headspace concentrations of about 10 ppm with 10 ml of medium and a headspace of 150 cm^3^, equivalent to only about 60 nmol total, CO-coupled perchlorate reduction could not be observed directly by analyses of changes in perchlorate concentrations in hypersaline media. Under the assay conditions, complete CO oxidation would have resulted in a maximum perchlorate decrease of only 1.5 μM out of 0.01 M assuming the following stoichiometries with chlorite as the end-product:

$$\begin{array}{c} \mathrm{CO}\;+\;{C1O}_{4}^{-}\to {\mathrm{CO}}_{2}+\;{\mathrm{ClO}}_{3}^{-}\\ \mathrm{CO}\;+\;{\mathrm{ClO}}_{3}^{-}\to {\mathrm{CO}}_{2}\;+\;{\mathrm{ClO}}_{2}^{-} \end{array}$$

Therefore, coupling of CO oxidation to perchlorate was established by comparing CO uptake in anaerobic assays with no electron acceptors to uptake in aerobic assays (air headspaces), and anaerobic assays with perchlorate only (0.01 M), nitrate only (0.25 or 25 mM), and perchlorate (0.01 M) with a low concentration of nitrate (0.25 mM) as a *nar* gene inducer.

**FIGURE 1:**
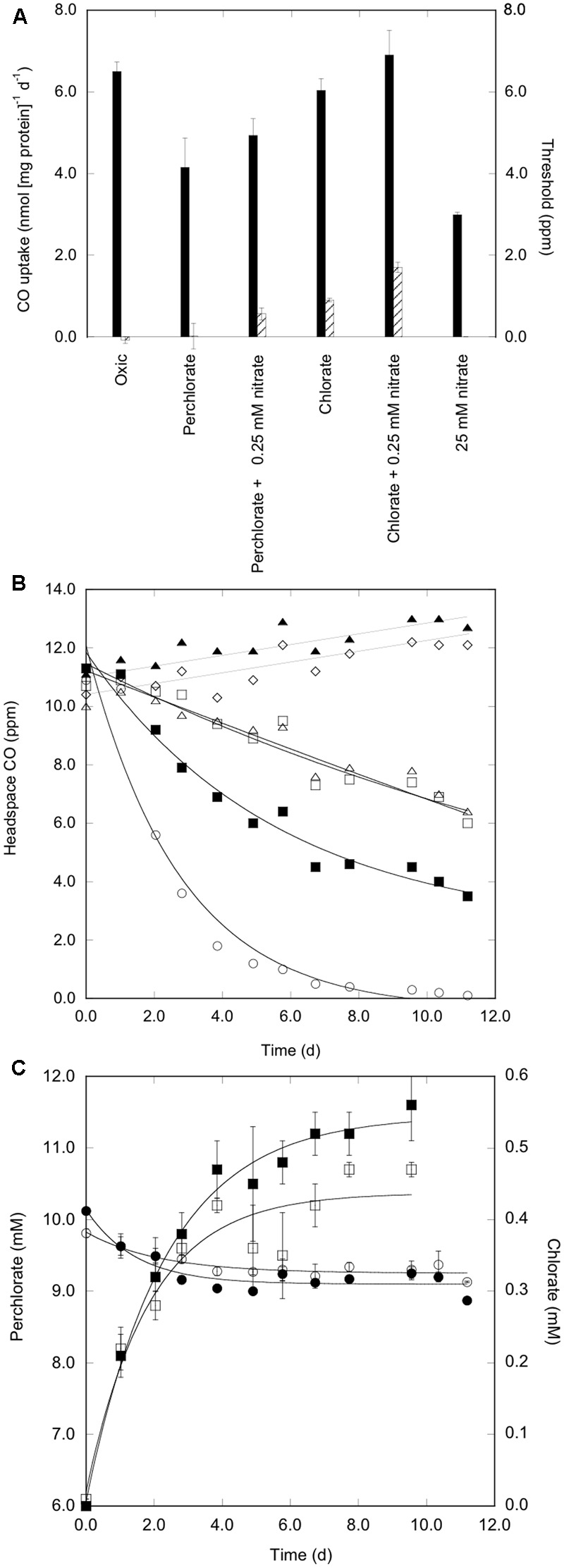
Perchlorate-coupled CO uptake by *Haloarcula* sp. PCN7. **(A)** CO uptake rates (nmol CO [mg protein]^-1^ d^-1^; solid bars) and uptake thresholds (ppm; cross-hatched bars) for isolate *Haloarcula* sp. PCN7 during varied oxic and anoxic incubations in 3.8 M NaCl. **(B)** Headspace CO versus time for isolate *Haloarcula* sp. PCN7 during varied oxic and anoxic incubations in 3.8 M NaCl; oxic, ○; perchlorate plus nitrate, ■; perchlorate, □; 25 mM nitrate, △; 0.25 mM nitrate, ▲; no electron acceptor, ♢. **(C)** Perchlorate uptake (circles) and chlorate production (squares) for perchlorate only (open symbols) and perchlorate plus nitrate (closed symbols) during assays with isolate *Haloarcula* sp. PCN7 in media with 2.5 mM pyruvate. All values are means of triplicate cell suspensions ± 1 standard error. Negative threshold values were not statistically different than zero.

Under these conditions, CO uptake occurred at rates of 3.0–6.5 nmol [mg protein]^-1^ d^-1^, but only in the presence of oxygen, perchlorate with or without 0.25 mM nitrate, or nitrate at 25 mM; no uptake was observed with 0.25 mM nitrate alone, without electron acceptors or in killed controls (**Figures 1A,B**). In the low nitrate treatment, nitrate was likely unavailable for CO oxidation due to rapid consumption during basal metabolism. Initial CO uptake rates in perchlorate and nitrate treatments were similar, and significantly lower than rates in aerobic assays (**Figures 1A,B**). Although treatments with 0.01 M perchlorate plus 0.25 mM nitrate yielded a small increase in uptake relative to perchlorate only (**Figure 1A**), the effect was not significant and varied among trials. These observations differ from those for *Hfx*. *mediterranei* 33500^T^, which reduced perchlorate and chlorate only after growth in anaerobic media with nitrate (Martinez-Espinosa et al., 2015). This indicates that synthesis of dissimilatory nitrate reductase might be induced by anoxia alone in some denitrifying extreme halophiles as has been observed for some bacteria [e.g., *Achromobacter cycloclastes* (Coyne and Tiedje, 1990)]. Although nitrate is likely present in Mars’ regolith (Kounaves et al., 2014; Stern et al., 2017), results from *Haloarcula* sp. PCN7 suggest that it might not be required to initiate perchlorate reduction in brines.

Threshold levels for CO uptake did not vary consistently among aerobic, perchlorate, and nitrate treatments (**Figure 1A**). Nonetheless, CO uptake with each of the electron acceptors continued at levels comparable to or less than ambient terrestrial atmospheric values (0.2–0.4 ppm, about 0.02–0.04 Pa), which are far below those in Mars’ atmosphere. This suggests that CO uptake need not be confined to Mars’ surface regolith, but that it could occur in sub-surface horizons that might provide more favorable physical conditions (e.g., lower UV/ionizing radiation exposure).

Results from a separate assay were similar, but by including 2.5 mM pyruvate in the incubation medium as catabolic substrate, it was possible to observe perchlorate reduction and chlorate formation (**Figure 1C**). During the period of maximum CO oxidation, perchlorate decreased by approximately 1 mM, while chlorate increased by about 0.5 mM (**Figure 1C**); chlorite formation from chlorate might have accounted for part or all of the difference. The decrease in perchlorate and increase in chlorate concentrations were greatest in the presence of 0.25 mM nitrate (**Figure 1C**), but perchlorate reduction did not require nitrate, which was consistent with results from CO oxidation. The fact that CO uptake did not occur in the absence of an electron acceptor (e.g., perchlorate or 25 mM nitrate), but occurred while perchlorate was reduced and chlorate was formed confirmed the potential for CO-coupled perchlorate reduction by extreme halophiles.

### Chlorate-Coupled CO Oxidation

Carbon monoxide uptake was also observed with chlorate, an intermediate in the perchlorate reduction pathway (Coates and Achenbach, 2004). CO uptake rates with chlorate were comparable to uptake rates with oxygen and exceeded uptake rates with perchlorate (**Figure 1A**), a pattern consistent with observations of chlorate and perchlorate reduction by *Hfx*. *mediterranei* 35000^T^ (Martinez-Espinosa et al., 2015). However, CO uptake threshold values with chlorate were significantly higher than with other oxidants (**Figure 1A**), which indicated that the relative abundance of perchlorate and chlorate could affect CO uptake rates when both co-occur. This possibility was confirmed by comparing results from assays with 9:1 and 1:9 mM perchlorate:chlorate ratios, respectively (**Table 1** and Supplementary Figure 3). In agreement with prior assays, CO uptake rate constants with 9:1 mM chlorate:perchlorate were greater than uptake rate constants with 1:9 mM perchlorate:chlorate. On the other hand, the addition of 1 mM chlorate to 9 mM perchlorate yielded CO uptake thresholds (1.67 ppm) substantially greater than values observed for perchlorate alone or perchlorate plus 0.25 mM nitrate; thresholds were even higher with 9 mM chlorate plus 1 mM perchlorate (**Table 1**). Nonetheless, all uptake threshold values were lower than ambient atmospheric levels on Mars, and thus would permit surface and sub-surface CO uptake.

**Table 1:** CO uptake rate constants (d^-1^) and uptake thresholds (ppm) for *Haloarcula* sp.

Treatment	Rate constant	Threshold
Aerobic	0.506 (0.034)^a^	0.08 (0.05)^a^
9:1 perchlorate:chlorate	0.389 (0.009)^b^	1.67 (0.11)^b^
1:9 perchlorate:chlorate	0.568 (0.023)^a^	3.75 (0.25)^c^

*PCN7 incubated under aerobic conditions or with anoxic conditions and varied ratios of perchlorate and chlorate concentrations, concentrations in millimolar. All values are means (±1 standard error) of triplicate determinations. Statistically significant differences based on analysis of variance with a Bonferroni *post hoc* test are indicated by superscripts.*

### Response to Elevated Perchlorate Concentrations

**FIGURE 2:**
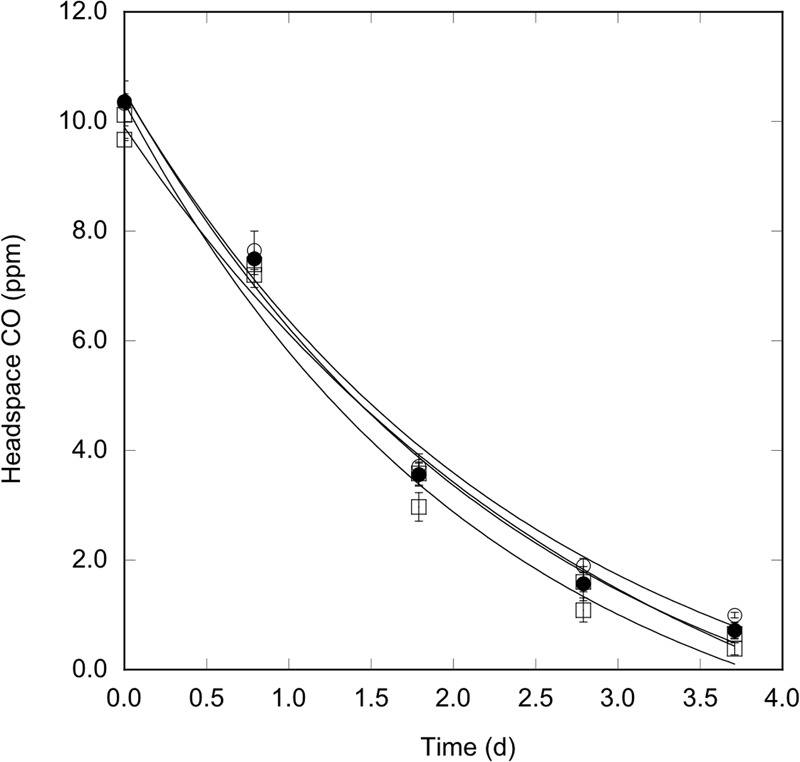
Perchlorate tolerance by *Haloarcula* sp. PCN7 under oxic conditions. CO uptake by *Haloarcula* sp. PCN7 during aerobic incubations with no perchlorate (○) or 0.01 (●), 0.1 (□), or 1 M (■) perchlorate. All values are means of triplicate cell suspensions for ±1 standard error.

Although CO uptake assays were typically conducted with 0.01 M perchlorate, additional assays explored activity at concentrations up to 1 M. No differences in CO uptake rate constants or thresholds were observed when *Haloarcula* sp. PCN7 was incubated with ambient air and 0–1 M perchlorate in a brine medium with a water potential of -19 MPa (**Figure 2**). Oxic incubations facilitated comparisons with some prior studies. For example, Oren et al. (2014) found no effect of 0.2 M perchlorate on aerobic growth by several archaeal extreme halophiles, but noted partial to substantial inhibition at higher concentrations (0.4–0.6 M). Limited tolerance of 1 M perchlorate has been also reported for several bacterial isolates during aerobic growth assays (Al Soudi et al., 2017), but those assays were conducted at much higher water potentials (-1.4 to -5.6 MPa, e.g., moderate salt concentrations and less physiological water stress) than used in this study (-19 MPa). Thus, the ability to oxidize CO during substantial water stress (i.e., lower water potentials) and simultaneously high concentrations of a potent chaotrope [perchlorate (Cray et al., 2013)] is unprecedented.

**FIGURE 3:**
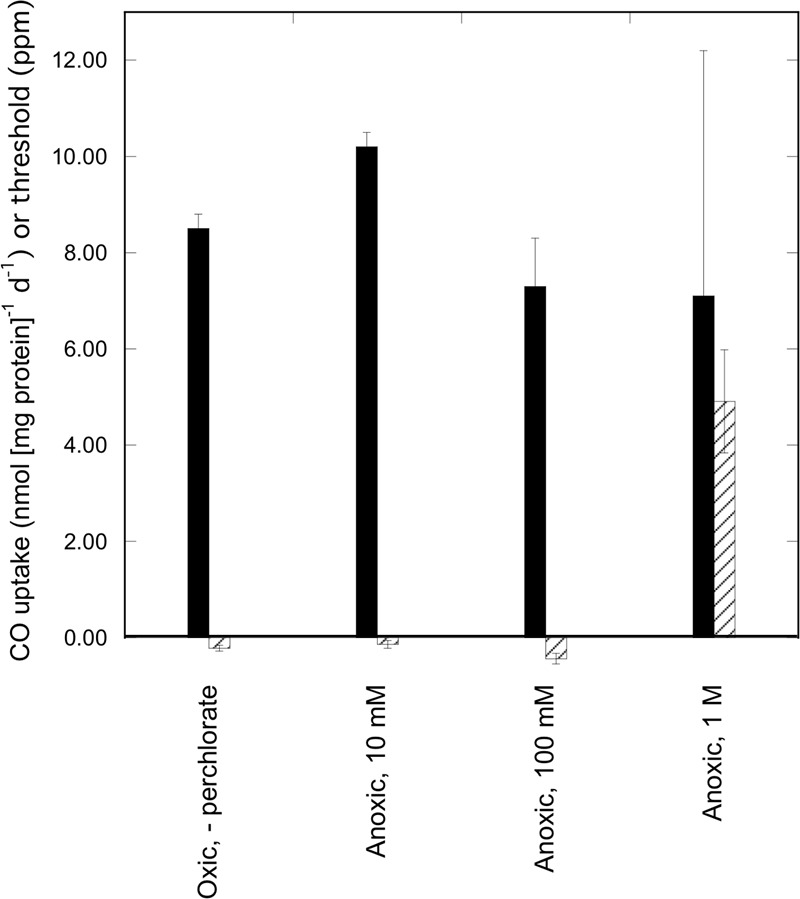
Perchlorate tolerance by *Haloarcula* sp. PCN7 under anoxic conditions. CO uptake rates (nmol CO [mg protein]^-1^ d^-1^; solid bars) and uptake thresholds (ppm; cross-hatched bars) for isolate *Haloarcula* sp. PCN7 during aerobic and anaerobic incubations in 3.8 M NaCl with varied concentrations of perchlorate; values are means of triplicate cell suspensions ± 1 standard error; negative threshold values were not significantly different than zero.

*Haloarcula* sp. PCN7 was also able to couple CO uptake with elevated perchlorate concentrations (up to 1 M) under anaerobic conditions. Anaerobic perchlorate tolerance at such high levels has not been previously documented, although 0.05 M perchlorate did not affect CO oxidation by *Alkalilimnicola ehrlichii* MLHE-1 (King, 2015), which was unable to use it as an electron acceptor. For *Haloarcula* sp. PCN7 CO uptake rates for 0.01, 0.1, and 1 M treatments did not differ statistically, but uptake rates with 1 M perchlorate were more variable (**Figure 3**) and threshold concentrations were significantly higher (4.9 ± 1.0 ppm) than for 0.01 and 0.1 M perchlorate (not statistically different than zero). Differences in thresholds with 1 M perchlorate could reflect lower water potentials in the assay media (-24.5, -25.4, and -32.6 MPa for 0.01, 0.1, and 1 M, respectively). Collectively, these results indicated that perchlorate was not only tolerated at high concentrations under anaerobic conditions, but that it was exploited as an oxidant. This in turn supports the possibility that high perchlorate concentrations in extraterrestrial brines could sustain microbial life, potentially including biotypes specifically adapted for perchlorate dissimilation.

### Perchlorate–CO Coupling by Nitrate-Respiring Extreme Halophiles

**FIGURE 4:**
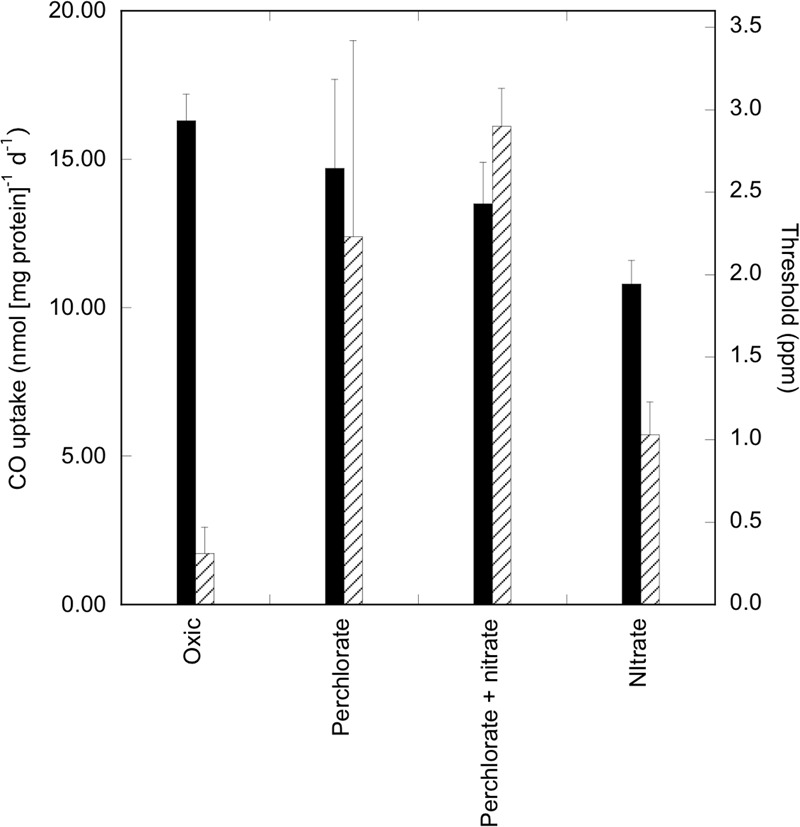
Perchlorate-coupled CO uptake by *Halobaculum* sp. WSA2. CO uptake rates (nmol CO [mg protein]^-1^ d^-1^) and uptake thresholds (ppm) for isolate *Halobaculum* sp. WSA2 during aerobic and anaerobic incubations in 3.8 M NaCl with perchlorate, perchlorate + 0.25 mM nitrate, or with 25 mM nitrate; values are means of triplicate cell suspensions ±1 standard error.

Coupling of CO oxidation with perchlorate reduction was confirmed with a second isolate, *Halobaculum* sp. WSA2. Like *Haloarcula* sp. PCN7, *Halobaculum* sp. WSA2 possesses a form I molybdenum-dependent CODH and a dissimilatory nitrate reductase (Supplementary Figures 1, 2). However, unlike *Haloarcula* sp. PCN7, *Halobaculum* sp. WSA2 produces nitrite as a terminal product from nitrate reduction, and does not possess genes for nitrite or nitrous oxide reduction based on initial results of a genome analysis. It oxidized CO under aerobic conditions, or with nitrate, perchlorate, or perchlorate plus nitrate at rates similar to those for *Haloarcula* sp. PCN7 and with similar threshold values (**Figure 4**).

The ability of *Halobaculum* sp. WSA2 to reduce perchlorate is notable, because a nitrate-respiring gammaproteobacterium, *A*. *ehrlichii* MLHE-1, could not do so although it readily oxidized CO with nitrate (King, 2015). This might reflect a fundamental difference in the location of the nitrate reductase active site in the narGH enzyme system of haloarchaea versus that of some bacteria (Martinez-Espinosa et al., 2007). In particular, the haloarchaeal narG active site has been described as periplasmic (Martinez-Espinosa et al., 2007), which could result in greater accessibility to perchlorate than a cytoplasmic orientation in bacteria does. Thus, only a sub-set of CO-oxidizing nitrate respirers may reduce perchlorate, and a similar constraint might apply to denitrifiers.

In summary, this study demonstrates for the first time that perchlorate and chlorate can be coupled as electron acceptors to anaerobic CO oxidation in NaCl brines by diverse euryarchaeal extreme halophiles capable of denitrification or nitrate respiration. Under anoxic conditions, perchlorate concentrations from 0.01 to 1 M in 3.8 M NaCl with or without nitrate supported CO oxidation. Oxidation occurred at CO concentrations comparable to those in Mars’ atmosphere and at concentrations comparable to or lower than those in Earth’s atmosphere.

The ubiquity of CO in the cosmos (Dickman, 1978), its presence at relatively high concentrations in the solar system (Elsila et al., 1997; Greaves et al., 2011; Materese et al., 2015), and the possibility of coupling CO oxidation to diverse electron acceptors (Ragsdale, 2004; King, 2006; King and Weber, 2007) suggest that CO-based metabolism could fuel microbial activity in some of the exoplanetary systems that have been discovered to date. Evidence for high perchlorate concentrations on Mars and results from this study also indicate that CO could fuel metabolism by either relict or introduced extreme halophiles in hydrated salts or brines (Ojha et al., 2015).

## Author Contributions

MM isolated and characterized strains, conducted growth, CO, and chemical assays, and contributed to manuscript development. GK conceived the study, conducted fieldwork and sample collections for isolate enrichments, contributed to experimental analyses, and wrote the manuscript.

## Conflict of Interest Statement

The authors declare that the research was conducted in the absence of any commercial or financial relationships that could be construed as a potential conflict of interest.

## Footnotes

1 https://img.jgi.doe.gov
