# Addendum: Perchlorate-Coupled Carbon Monoxide (CO) Oxidation: Evidence for a Plausible Microbe-Mediated Reaction in Martian Brines

**DOI:** 10.3389/fmicb.2019.01158

**Published:** 2019-05-29

**Authors:** Marisa R. Myers, Gary M. King

**Affiliations:** Department of Biological Sciences, Louisiana State University, Baton Rouge, LA, United States

**Keywords:** carbon monoxide, extreme halophile, perchlorate reduction, Mars, chlorate reduction

Myers and King (2017) described perchlorate-coupled carbon monoxide oxidation by two haloarchaeal isolates that were presumed to be monocultures, *Halobaculum* sp. WSA2 and *Haloarcula* sp. PCN7. Although both isolates had been subjected to a battery of standard microbiological analyses, results from genome sequence analyses indicated that the former isolate was axenic while the latter was mixed. Subsequent purification and characterization efforts yielded an axenic isolate, *Halobacterium* sp. PCN9 and a purified *Haloarcula* sp. PCN7. *Halobacterium* sp. PCN9 oxidized CO and reduced perchlorate, and was shown to account for the results originally reported by Myers and King (2017).

*Halobaculum* sp. WSA2 was identified as a nitrate-respiring (denitratating, reducing nitrate to nitrite) CO oxidizer on the basis of a single 16S rRNA gene sequence deposited with NCBI as MF767889; it was most closely related to *Halobaculum roseum* D90^T^ (98.19% identity). *Haloarcula* sp. PCN7, was identified as a denitrifying (respiring nitrate to dinitrogen) CO oxidizer that yielded two distinct 16S rRNA gene sequences during PCR amplification as described by McDuff et al. (2016). These two sequences (deposited with NCBI as MK490920 and MK490921) were most closely related to the 16S rRNA gene sequence of *Haloarcula taiwanensis* and *Haloarcula japonica* (99.37% and 99.45% identity, respectively) based on BLAST analyses. Since halobacterial genomes often contain multiple 16S rRNA genes (e.g., Cui et al., 2009), the presence of these two sequences was not considered unusual.

However, genomic analyses of paired-end DNA sequences generated on an Illumina Miseq using 2 × 250 bp chemistry at Michigan State University subsequent to the work reported by Myers and King (2017) revealed that the outcome of enriching *Haloarcula* sp. PCN7 was a mixed culture, while *Halobaculum* sp. WSA2 was confirmed to be a pure culture (IMG Metagenome/Genome IDs 3300032142 and 2756170195, respectively). For the mixed culture, two sets of contigs were evident after read assembly and annotation. One set included the two 16S rRNA genes along with numerous other genes that were most closely related to *Haloarcula*. Notably, these contigs did not contain genes essential for denitrification (e.g., nitric oxide and nitrous oxide reductase genes), or CO oxidation (e.g., form I CO dehydrogenase genes).

A second set of contigs was comprised of genes most closely related to *Halobacterium*, including a 16S rRNA gene that was 98.66% identical to *Halobacterium noricense* (available as Ga0334598_104419 from IMG metagenome ID 3300032142). In addition, the *Halobacterium* contigs contained genes essential for CO oxidation (e.g., *coxL* available as Ga0334598_11585 from IMG metagenome ID 3300032142).

Additional transfers of the original mixed culture ultimately yielded two distinct isolates. *Haloarcula* sp. PCN7 was obtained as a monoculture that was morphologically indistinguishable from the original mixed culture, but neither oxidized CO nor denitrified, a finding consistent with results from genome sequence annotation. Absence of *Halobacterium* from this derivative of the original mixed culture was supported by results from a PCR analysis (Figure 1) using primers designed for the *Halobacterium* 16S rRNA gene sequence identified by genome analysis (HalobacF: 5′-ATGCTAGTTGTGCGGGTTCA−3′ and reverse primer HalobacR:. 5′-CGAGCAGATTTCCCAACGGA−3′). PCR with these primers was positive for the original mixed culture, positive for a *Halobacterium* isolate derived from it, and negative for the *Haloarcula* sp. PCN7 monoculture (Figure 1). *Haloarcula* sp. PCN7 has been deposited as an axenic culture with the Deutsche Sammlung von Mikroorganismen und Zellculturen (DSM 103645).

**Figure 1:**
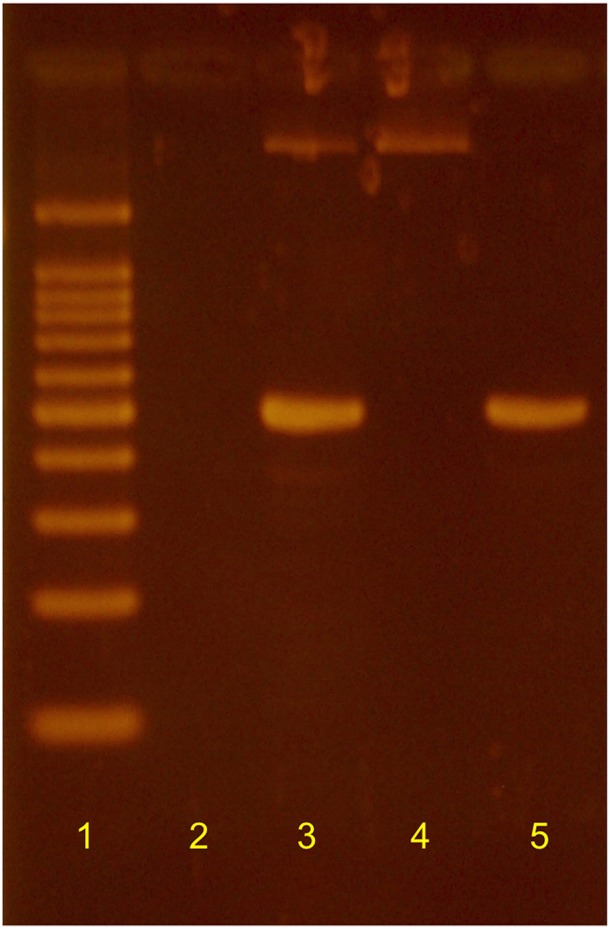
Results of PCR using *Halobacterium*-specific primers based on the 16S rRNA gene sequence of *Halobacterium* sp. PCN9. Lane 1: l00 bp ladder; 2: negative control; 3: *Halobacterium* sp. PCN9 monoculture; 4: *Haloarcula* sp. PCN7 monoculture; 5: *Haloarcula* sp. PCN7 mixed culture as used in original analyses of CO-coupled perchlorate reduction (Myers and King, 2017).

The second isolate in the original mixed culture was identified as a member of the genus *Halobacterium*, and designated as *Halobacterium* sp. PCN9. This isolate has been deposited with the American Type Culture Collection as TSD-126 and with the Belgian Coordinated Collection of Microorganisms as LMG 31022. The 16S rRNA sequence for *Halobacterium* sp. PCN9 was identical to the *Halobacterium* sequence found during genomic analysis of the original mixed *Haloarcula* sp. PCN7 culture. The genome of *Halobacterium* sp. PCN9 also harbored genes for CO oxidation as indicated by successful amplification of the *coxL* gene as described by McDuff et al. (2016); the sequence for this gene (Genbank accession MK262901) was identical to that observed in the mixed culture.

*Halobacterium* sp. PCN9 oxidized CO under oxic conditions as described by Myers and King (2017) in contrast to the *Haloarcula* sp. PCN7 monoculture (Figure 2A). CO uptake assays were also conducted with 10 mM perchlorate as an electron acceptor under anoxic conditions as described by Myers and King (2017). *Halobacterium* sp. PCN9 reduced perchlorate to chlorate (Figure 3) while simultaneously oxidizing CO (Figure 2B) as described in Myers and King (2017). Although the *Haloarcula* sp. PCN7 monoculture could not oxidize CO (Figure 2A), a 1:1 mixture (based on A_600_) of *Halobacterium* sp. PCN9 and the *Haloarcula* sp. PCN7 monoculture oxidized CO, reduced perchlorate, and produced chlorate as reported by McDuff et al. (2016) (Figures 2B, 3).

**Figure 2:**
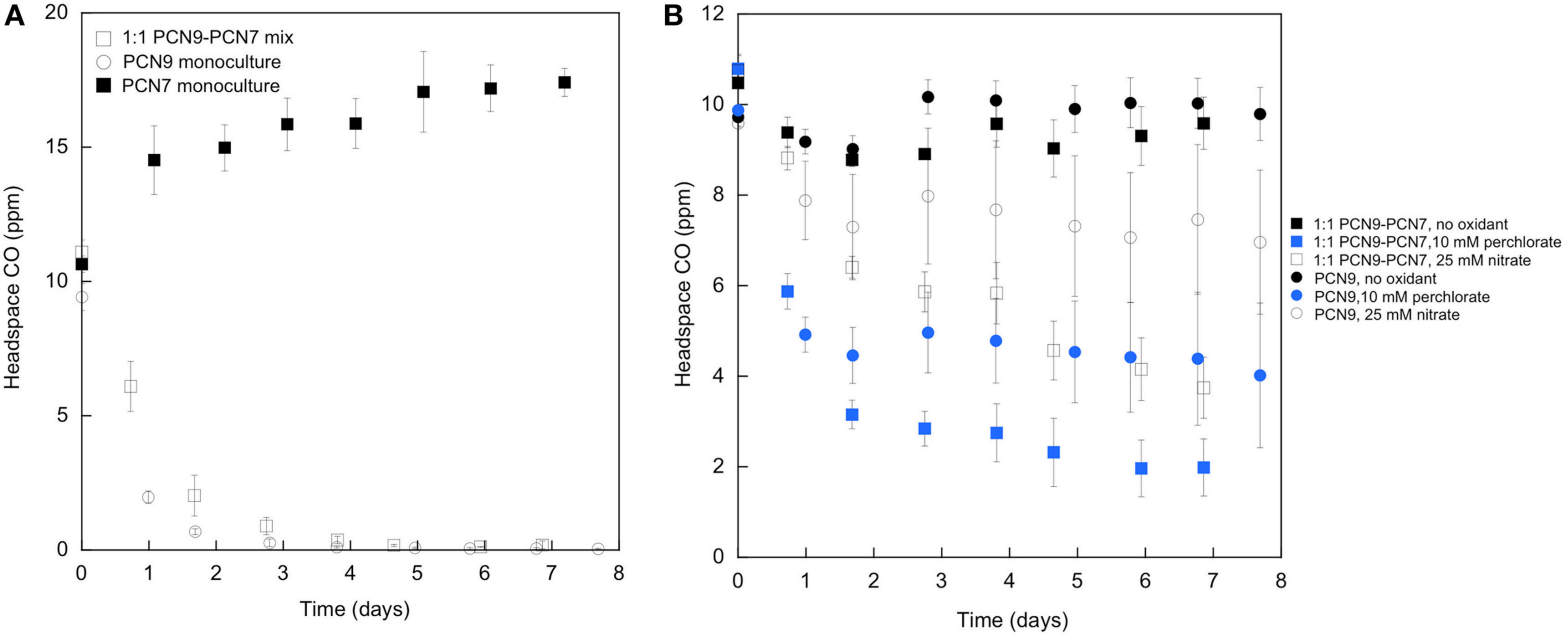
**(A)** CO uptake under oxic conditions by monocultures of *Halobacterium* sp. PCN9 and *Haloarcula* sp. PCN7, and a 1:1 mixed culture of both. **(B)** CO uptake under anoxic conditions by monocultures of *Halobacterium* sp. PCN9 and *Haloarcula* sp. PCN7, and a 1:1 mixed culture of both with and without perchlorate. Assays as described by Myers and King (2017).

**Figure 3:**
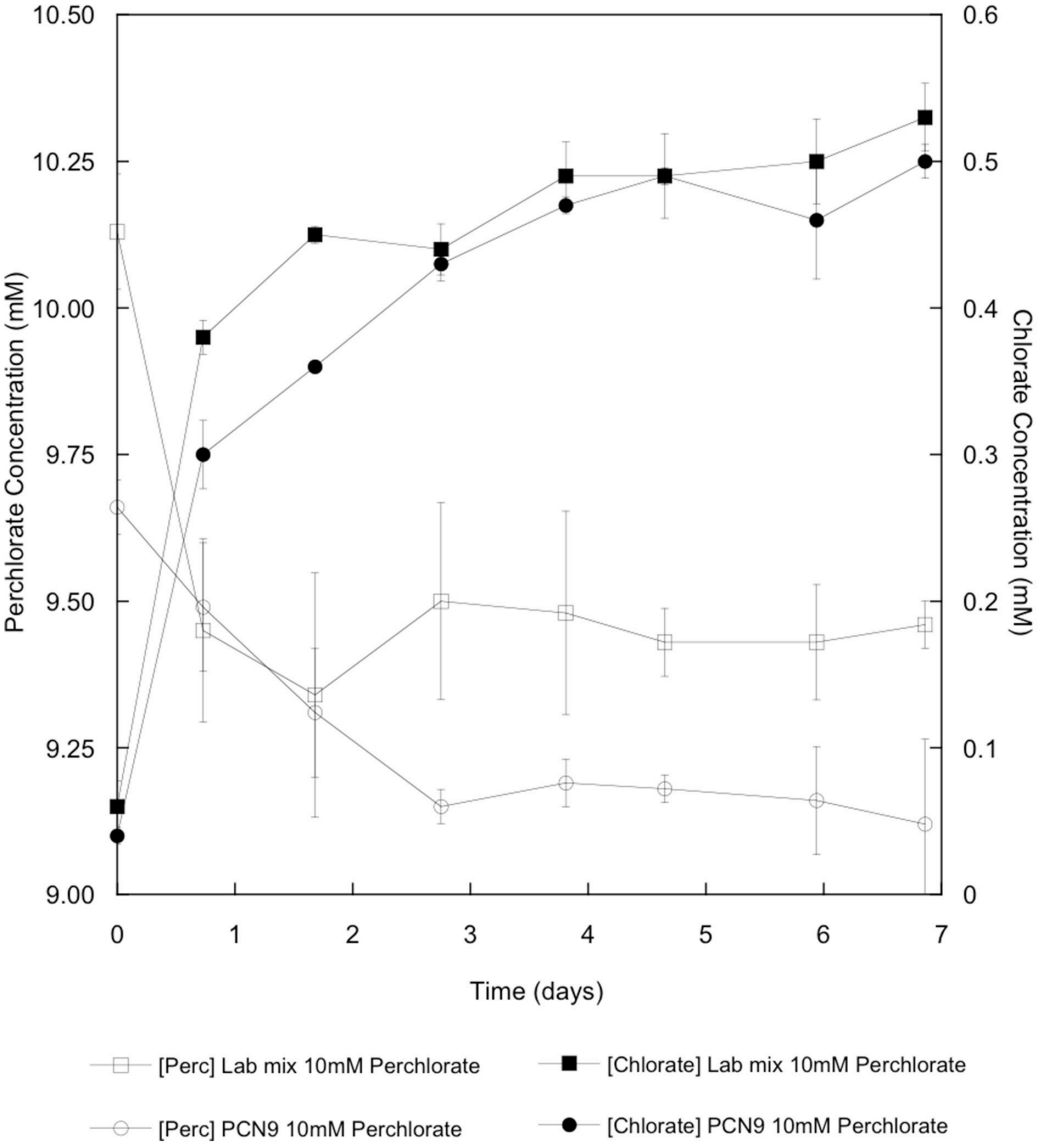
Perchlorate reduction (open symbols) and chlorate accumulation (closed symbols) for a monoculture of *Halobacterium* sp. PCN9 and a 1:1 mixed culture of *Halobacterium* sp. PCN9 and *Haloarcula* sp. PCN7 incubated anaerobically with conditions and assays as described by Myers and King (2017).

These results confirmed observations by Myers and King (2017) that extreme halophiles can couple CO oxidation to perchlorate reduction, but they revealed that a member of the genus *Halobacterium* (*Halobacterium* sp. PCN9) rather *Haloarcula* sp. PCN7 was responsible for the reported activity. In addition, analysis of the *Halobaculum* sp. WSA2 genome sequence confirmed that it was a monoculture, and thus responsible for CO-coupled perchlorate reduction as described by Myers and King (2017).

## References

[B1] Cui H.-L. Zhou P.-J. Oren A. Liu S.-Jd. (2009). Intraspecific polymorphism of 16S rRNA genes in two archaeal genera, *Haloarcula* and *Halomicrobium*. Extremophiles 13, 31–37. 10.1007/s00792-008-0194-2 18836684

[B2] McDuff S. King G. M. Neupane S. Myers M. R. (2016). Isolation and characterization of extremely halophilic CO-oxidizing Euryarchaeota from hypersaline cinder, sediments and soils and description of a novel CO oxidizer, *Haloferax namakaokahaiae* Mke2.3. FEMS Microbiol. Ecol. 92:28 10.1093/femsec/fiw028 26906098

[B3] Myers M. R. King G. M. (2017). Perchlorate-coupled carbon monoxide (CO) oxidation: evidence for a plausible microbe-mediated reaction in martian brines. Front. Microbiol. 2017:2571 10.3389/fmicb.2017.02571 PMC5743682 29312249

