# Supplementary material for: Perchlorate-Coupled Carbon Monoxide (CO) Oxidation: Evidence for a Plausible Microbe-Mediated Reaction in Martian Brines
Source: Front Microbiol. 2017 Dec 22;8:2571. doi: 10.3389/fmicb.2017.02571 (PMC5743682; doi:10.3389/fmicb.2017.02571)

Myer and King, Supplementary Materials


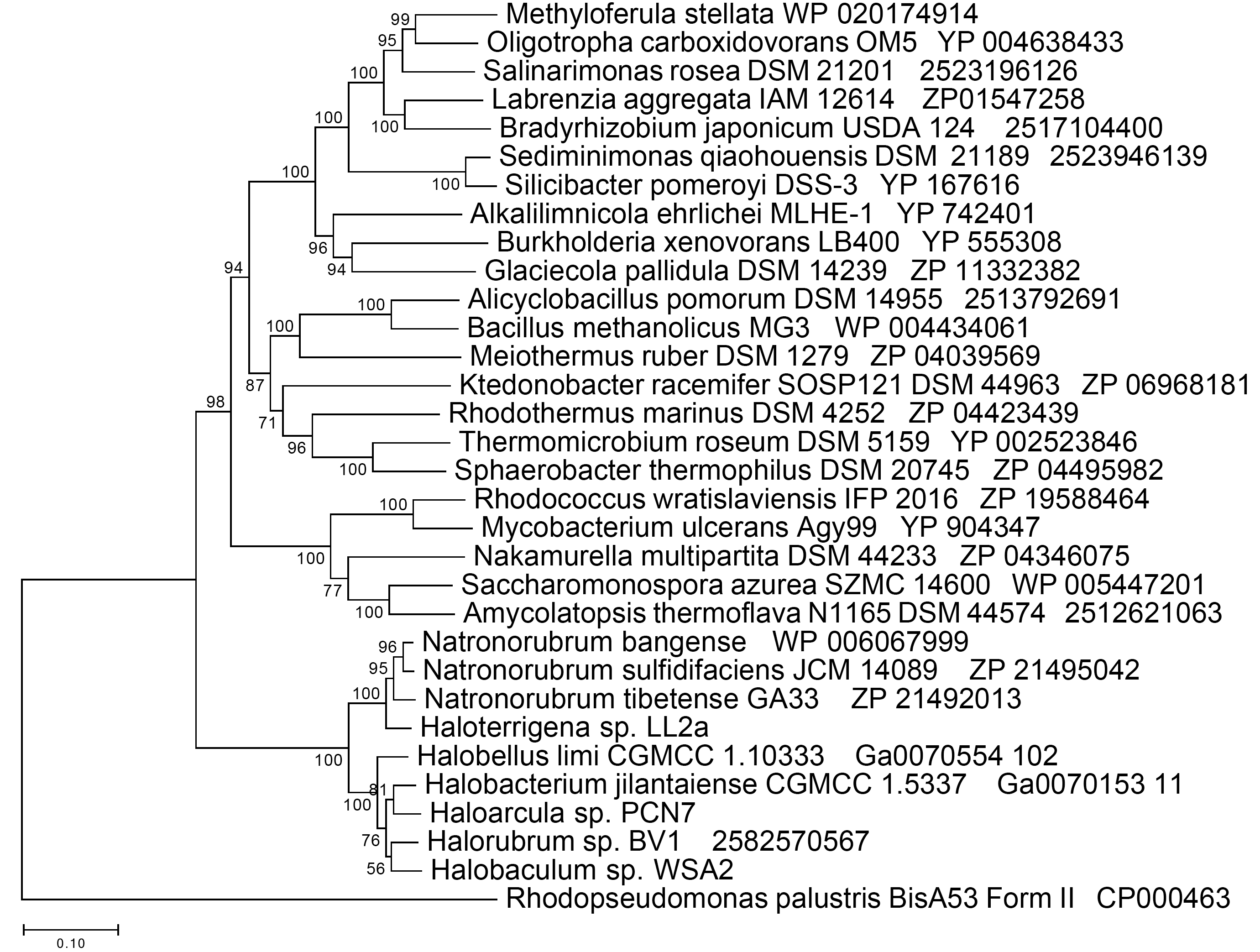
Supplementary Figure 1. Evolutionary relationships of *coxL* obtained using a neighbor-joining method with a Poisson correction. Gapped and missing data were eliminated; a total of 718 positions were included in the final dataset; distances are in units of amino acid substitutions per site. Numbers at branches indicate bootstrap support > 70%. Arrows indicate isolates used in this study.Supplementary Figure 2. Evolutionary relationships of *narG* obtained using a neighbor-joining method based on a Poisson correction. Gapped and missing data were eliminated; a total of 717 positions were included in the final dataset; distances are in units of amino acid substitutions per site. Numbers at branches indicate bootstrap support > 70%. Arrows indicate isolates used in this study.


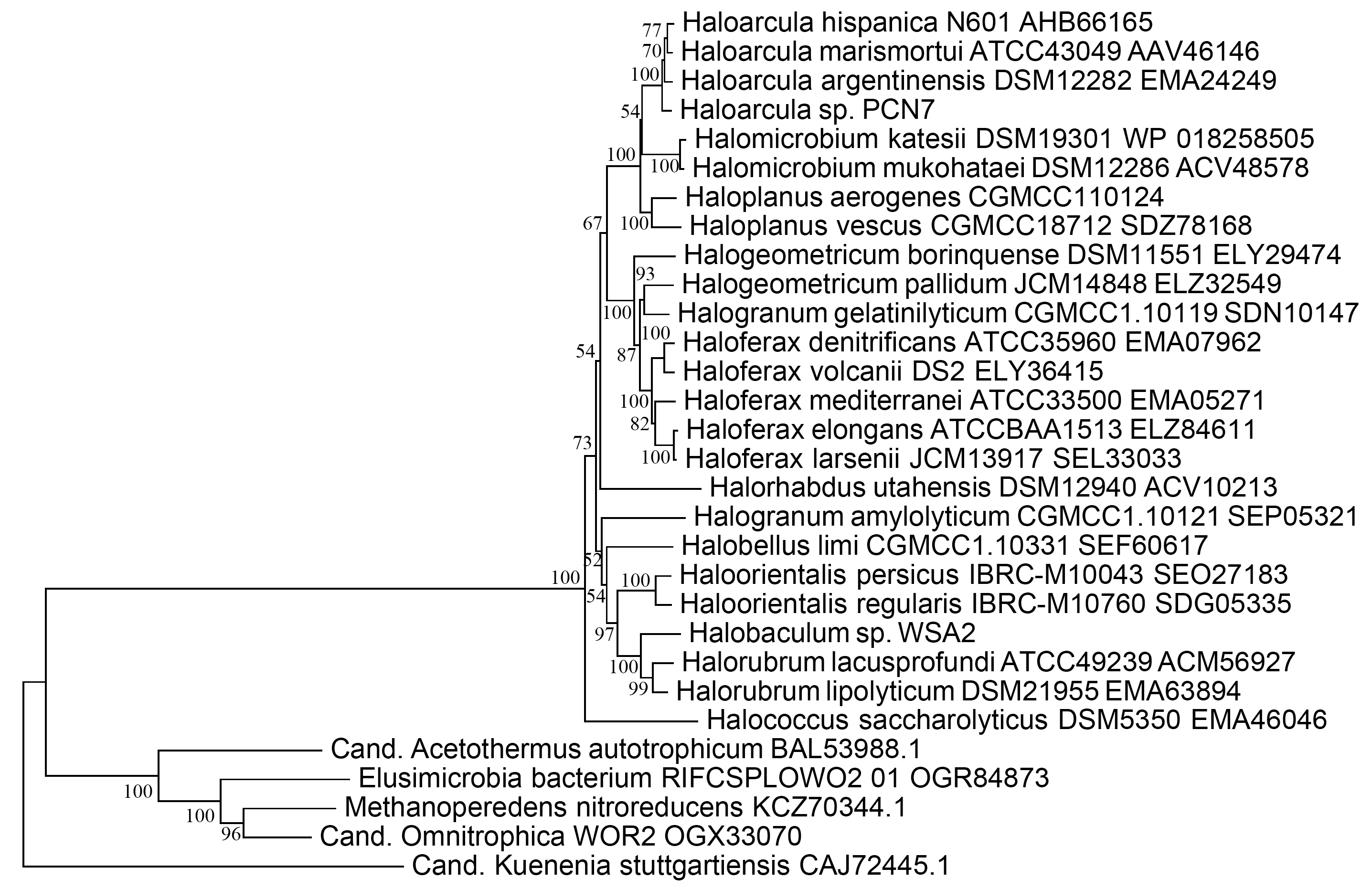


Supplementary Figure 3. CO uptake by *Haloarcula* sp. PCN7 during aerobic incubations with no perchlorate (  ) or anaerobic incubations with 9 mM perchlorate + 1 mM chlorate ( ), 1 mM perchlorate + 9 mM chlorate (🞏) or with no electron acceptor () perchlorate. All values are means of triplicate determinations ± 1 standard error
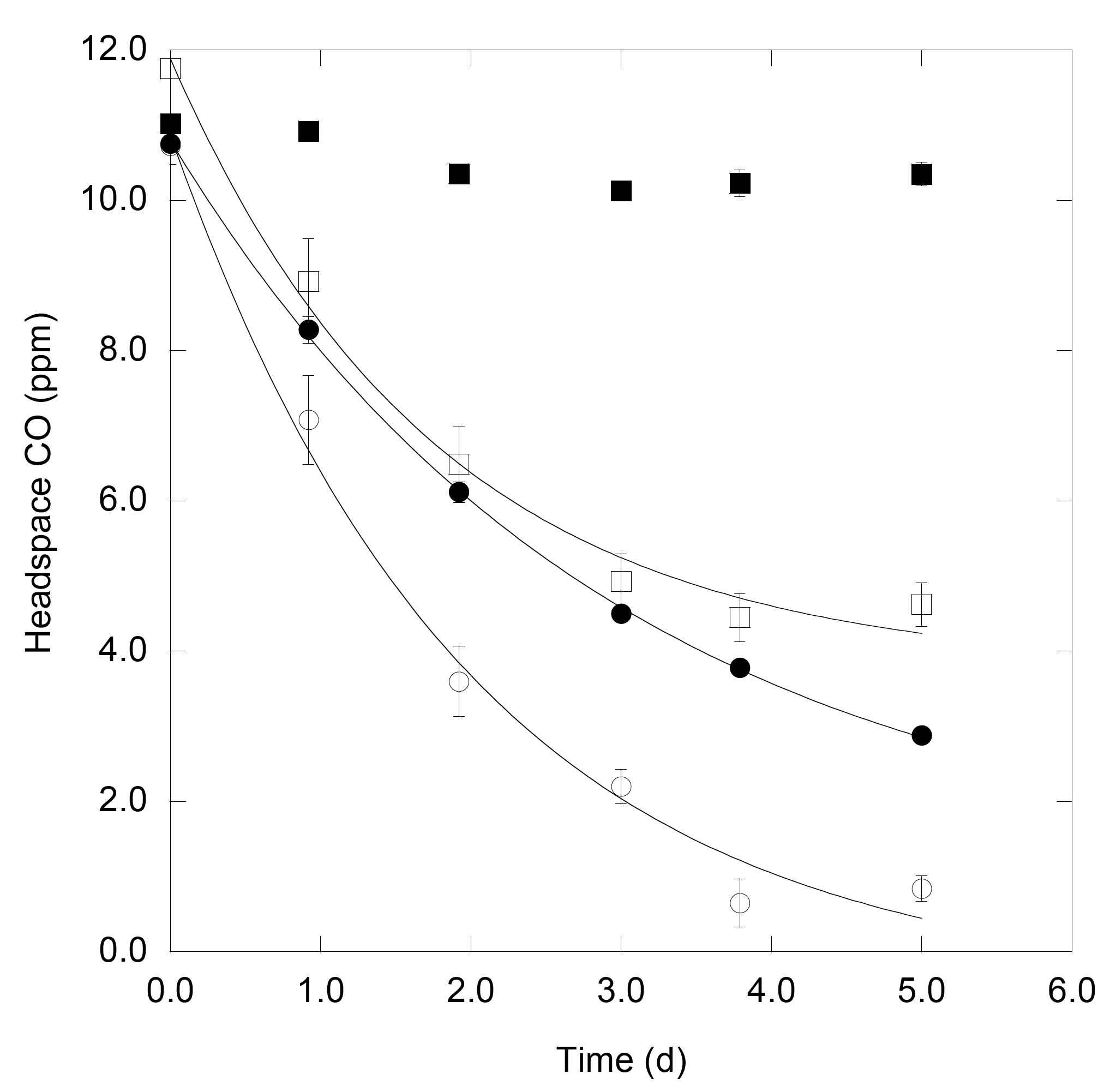

Supplement: Supplementary file 1 [file Data_Sheet_1.DOCX]
